# Aperiodic Dynamics of Cell Assemblies Recruited for L1 and L2 Processing of French *Wh*-Dependencies Highlight a Temporo-Parietal Engagement in Syntax

**DOI:** 10.3390/brainsci16060645

**Published:** 2026-06-17

**Authors:** Laurent Dekydtspotter, A. Kate Miller, Mike Iverson, Jih-Ho Cha, Ludan Yang, Jane A. Gilbert, Hongyu Zhang, Kent Meinert, Qin Li, Jae Hyun Ahn

**Affiliations:** 1 Department of French & Italian, Indiana University Bloomington, Bloomington, IN 47405, USA; janegilb@iu.edu; 2 Department of Linguistics, Indiana University Bloomington, Bloomington, IN 47405, USAludyang@iu.edu (L.Y.); qli3@iu.edu (Q.L.);; 3 Department of World Languages and Cultures, Indiana University Indianapolis, Indianapolis, IN 46202, USA; am27@iu.edu; 4 School of Health and Rehabilitation Sciences, University of Queensland, Brisbane, QLD 4067, Australia

**Keywords:** Merge, movement, language faculty, EEG, biolinguistics, bilingualism

## Abstract

**Highlights:**

**What are the main findings?**

- Aperiodic activity (exponents) in filler-gap processing during reading revealed differences between temporo-parietal and frontal areas across L1 and L2.
- Aperiodic activity across offsets and exponents in filler-gap processing during reading revealed some differences between L1 speakers and advanced L2 learners.

**What are the implications of the main findings?**

- Temporo-parietal engagement is consistent with a potential subnetwork dedicated to syntactic computations across L1 and L2.
- Aperiodic activity across L1 and L2 aligns with current theoretical understanding of syntax, favoring language-ready views of the biology of language.

**Abstract:**

**Background/Objectives:** A current debate addresses where syntactic Merge primarily resides: the left-hemisphere posterior inferior frontal gyrus (IFG) or the temporo-parietal cortex. For proponents of the former, the temporo-parietal cortex supports more effortful processing; for the latter, the IFG supports integration and conflict resolution. We examine aperiodic activity in processing *wh*-filler-gap dependencies in French for evidence from network dynamics addressing engagement in syntax across L1 and L2. **Methods:** We extracted aperiodic activity 1/f components (considering offsets as a reflection of neuronal spiking and exponents as a reflection of excitatory–inhibitory balance) out of power spectrum density at 0.5–40 Hz across occipital and bilateral frontal and temporo-parietal regions of interest (ROIs) in reading. **Results:** Greater exponents arose in temporo-parietal than frontal ROIs in L1 and L2, with strong spiking and regulation suggested by greater offsets and exponents in the occipital ROI in L2—unlike L1—and with potential modulation by L1–L2 representation overlaps. These patterns suggest distributed cell assemblies for L1 and L2 processing. Increased regulation in temporo-parietal ROIs across L1 and L2 cell assemblies might suggest a structural function across temporo-parietal cortices in syntactic processing. **Conclusions:** Aperiodic activity reflecting connectivity in L1 and L2 processing supports distinct L1 and L2 cell assemblies, with L2 patterns suggesting potential overlap between L1 and L2 circuit modules. Greater exponents in bilateral temporo-parietal ROIs across L1 and L2 indicate increased regulation, supporting the engagement of lateralized temporo-parietal cortices in computations. These effects are discussed by considering advances in syntactic theory and the biology of language readiness.

## 1. Introduction

A long-standing body of behavioral research on second language (L2) acquisition in poverty-of-the-stimulus situations argues that knowledge of an L2, much like knowledge of a first language (L1), involves an implicit lexico-grammatical subsystem within the generative capacity for natural language [1,2,3,4,5] (among many others). From a neurolinguistic perspective, L1-based L2 acquisition within general language constraints seemingly involves the establishment of a neurofunctional subsystem for the L2 within a single adaptive network for language [6,7,8,9]. Neurocognitive evidence showing that bilingual aphasia patients are affected in both languages [10,11,12] aligns with the view that L1 and L2 involve the perisylvian language network with proficiency-modulated L2 vs. L1 activation thresholds for distinct but overlapping microlevel neurofunctional subsystems within a single network. Cognitively, L2 acquisition as implicit learning involves the (re)assembly of syntax-semantics (syn-sem) features into feature sets for basic novel lexico-grammatical elements [13,14,15] engaging with syntactic computations. The processing of L2 input thus requires a computational mechanism that enables the morphosyntactic mapping of linear (phonological) representations to hierarchical syn-sem structures. Such a mapping involves the setting of novel lexico-grammatical representations characterizable as relexification [16]. New morphosyntactic subsystems for L2 result in somewhat different event-related potential (ERP) N400-P600 profiles, with L2 speakers exhibiting N400 effects where L1 speakers might exhibit P600 effects [17] (but see [18,19] for N400-P600 differences found in a continuum in L1 speakers). Even in advanced L2 learners matching the L2 target, typological distance from the L1 yields later P600 structural effects [20], consistent with the increased complexity of L2 processing.

L2 and L1 knowledge is therefore expected to reflect distinct distributed cell assemblies for L2 vs. L1 involving novel circuit modules for new L2 lexico-grammatical representations, with their activation thresholds affecting performance [10]. Across L1 and L2, population-based circuit modules for neurofunctional lexico-grammatical elements are presumed to engage with neuron-based dedicated circuitry for syntactic computations. Novel population-based circuit modules for new L2 features and basic elements beyond the L1 emerge in target input processing through Hebbian learning as neurons fire and wire together [21]. In a parsing-to-learn model, syntactic computations—involving possible basic lexico-grammatical elements for the target input—constrain the endogenous recruitment of cells into representational circuit modules as blocks of neural circuitry [22]. L2 neurofunctional subsystems thus constitute distributed cell assemblies [23,24] recruited across the language network already hosting the L1 [6].

On this view, guided by linguistic theory, therefore, the larger language network hosts circuit modules for basic symbolic representations across domains in L1 and L2, together with subcircuits dedicated to syntactic computations. This interaction supports the language faculty in the broad sense (FLB). Within FLB, a language involves the merging of a set of lexico-grammatical elements in the computation of hierarchical trees mapping to linear strings. In current minimalist theory, hierarchical computations involve the recursive merging of complex and elementary units into trees. The recursive merging of elements relies on complementary functions enabling the composition of new units and the maintenance of their elements in recursive computations [25]. This computational mechanism constitutes the basic language faculty in a narrow sense (FLN) accounting for Universal Grammar (UG) as a single abstract computational mechanism across all languages [26]. Under this design, a computational subnetwork for abstract syntax allows a “language-ready” human brain [27] enabling a “fully linguistic brain” through the constrained recruitment of circuit modules to accommodate linguistic representations. Parsing allowing for the decomposition of the target language input therefore enables updates to the lexicon in implicit learning [28,29]. The FLN constraining the recruitment of circuit modules for L2 representations across the FLB therefore enables L2 acquisition in parsing target input.

In parsing to learn [28,29], language generation as an innate mechanism likely relies on an (epi)genetically determined specialized neuron-to-neuron computational circuitry in a Sherringtonian view. It also relies on population-based circuit modules for lexico-grammatical elements in a Hopfieldian view. This interaction accounts for the fact that “the lexicon does not exist in isolation from grammar” [30] (p. 361). Population-based modules for lexical elements account for the activation of distinct brain regions tied to intrinsic features [31]. They certainly also account for new concepts. Still, basic conceptual categories (e.g., entities, animacy, and events) across grammars plausibly involve specialized neurons implicated in perception and cognition. Specialized neurons for animacy, for instance, would account for specific necrotic lesions leading to losses in the naming and recognition of foods, plants, and animals [32]. Complementary interactions between classical symbolic and connectionist cognitive models have long been expected [33]. We argue that they can likely be assessed by examining neural aperiodic dynamics across the brain.

Neurocognitive research has long sought to locate the implementation of basic syntactic computations characterizable by FLN [34,35,36,37,38,39]. Considering the biology of language, Boeckx and Benítez-Burraco [27] argued for a “language-ready brain” (p. 1) echoing FLN—distinct from “the fully linguistic brain” (p. 1) echoing FLB—with a central role of the thalamus and cortico-subcortical connectivity in syntactic computations [40]. Cortico-subcortical connectivity aligns with Murphy’s Representation Operation Structure Encoding (ROSE) architecture for syntax [41,42] based on oscillatory dynamics, in which syntax implicates gamma/delta coupling as new units are assembled and structurally integrated. Addressing the activity of cortical sites, morphosyntactic processing has been tied to “temporal, parietal, and pre-frontal cortices, with abstract linguistic representations encoded in more distributed and higher-level activation patterns” [43] (p. 1). Despite a left-hemisphere specialization for language, computations in grammatical acquisition in monolinguals can implicate bilateral activity across low parietal lobules (BA40) and frontal gyrus (BA44) [44]. Bilateral parietal activity has also been tied to processing load increases in L1 Chinese relative clause processing in bilinguals [45].

According to Friederici [34,46,47], the implementation of basic syntax resides in the left-hemisphere posterior inferior frontal gyrus (pIFG), with the temporo-parietal cortex supporting the processing of complex structures (see also [36]). In contrast, Bornkessel-Schlesewsky and Schlesewsky argued that basic “linguistic processing per se only takes place in temporal and parietal regions, but not in frontal cortex” [48] (p. 67), with IFG tied to competing alternatives in ambiguity [49] and in cognitive control tasks [50]. This latter position is further supported by the decomposition of IFG as matching necessary steps in externalization [51], so that the frontal cortex is not tied to hierarchical computations. Addressing linearization and hierarchy in aphasia, Matchin and Hickock crucially argued that agrammatism is linked to morphosyntactic sequencing affected by lesions in IFG [37]. In contrast, paragrammatism, resulting in “sentence monsters” [52] and poor comprehension, is linked to basic syn-sem combinations affected by lesions in temporo-parietal sites. Matchin and Hickock therefore associated externalization with IFG and abstract structures with the posterior medial temporal gyrus. As Murphy noted, “persistent or severe language deficits in aphasia are common only in patients with extensive temporoparietal damage, not frontal damage” [41] (p. 4). Additionally, direct electrocortical stimulations to IFG interfere with morphosyntactic performance in language production [53], consistent with the IFG supporting language externalization. We argue for a computational mechanism dedicated to hierarchy across complementary temporo-parietal cortices and subcortical connections, with frontal connections tied to sequencing, morphological mapping, and linearization [51], considering advances in theoretical linguistics.

In Hebbian learning, “intrinsic excitability changes create ensembles, whereas synaptic plasticity could become important at later stages, perhaps during learning or consolidation” [22] (p. 881). Neurofunctional micro-subsystems for L2 presumably implicate L1-shared circuit modules for features pre-recruited in L1 acquisition with new endogenously activated “circuit modules with synchronous, or closely correlated, coordinated activity” [22] (p. 876) for new features and lexico-grammatical elements. Hence, in L2 acquisition, intrinsic excitability changes in neuronal firing first build cell ensembles for L2 within an already L1-consolidated language network. Then, neuronal connectivity is enhanced, and firing synchronicity is increased, by adding new dendritic branches in the rewiring of new neural circuit modules for basic representations. Novel synaptic connections are then refined in consolidation. Greater activity in L2 than L1 processing is expected as the L2 subsystem develops [7,8,9]. Examining L1 French-L2 English learners, Golestani et al. indeed showed stronger language-area-specific blood-oxygenation-level–dependent (BOLD) signals in L2, suggesting greater energy expenditure to activate “less well-tuned neural representations” [54] (p. 1038). These L1 vs. L2 differences diminished as L2 proficiency increased. In neurofunctional networks, excitatory circuits implement neuronal representations, while inhibitory circuits regulate them by preventing excessive firing and decrementing competing subcircuits. In structural processing of L1 and L2 input, excitatory and inhibitory balance in computational stabilization could therefore reveal the engagement of an FLN subnetwork across distinct neurofunctional subsystems for L1 and L2.

Aperiodic activity (1/f^x^) of the electroencephalographic (EEG) signal’s power spectrum density profile, as spectral power reduces and frequency increases, has been used to investigate the dynamics of the ongoing activity of the brain. Aperiodic activity involves two parameters: The offset value has been argued to reflect the overall shift in power across frequencies [55]. The exponent x has been argued to reflect the balance of postsynaptic and transmembrane activity for excitatory and inhibitory circuits [56,57,58,59], with a steeper slope indicating greater inhibitory signal [60]. Aperiodic activity shifts in early childhood [61,62,63,64] and in older adulthood [58,65,66], reflecting changes in brain circuits. Crucially, aperiodic activity also shifts in task processing [67] and has been tied to arousal [68], consciousness [69,70], and performance [71,72,73], reflecting close ties between aperiodic activity and functional connectivity [74]. In neurocognition, input processing in L2 acquisition induces the recruitment of endogenously activated cells into novel population-based circuit modules for L2 features that go beyond the preexisting L1-recruited circuit modules. Within a biolinguistics framework, innate evolution-based Sherringtonian circuits devoted to hierarchical computations—together with streams in externalization—constrain cell recruitment for basic morphological Hopfieldian neuro-representations in an adaptive language network. We might therefore expect aperiodic activity differences between regions of interest (ROIs) to reflect distinct engagements in language processing across L1 and L2. We might also expect aperiodic activity to reflect different distributed cell assemblies recruited for L1 and L2, including overlapping circuit modules for more versus less L1-like symbolic representations for L2.

In the current study, we use offsets and exponents reflecting neuronal spiking and inhibitory regulation to investigate the dynamics of cell assemblies in the processing of *wh*-filler-gap dependencies in French by L1-English L2-French vs. L1-French L2-English bilinguals. We investigate aperiodic activity in occipital and bilateral temporo-parietal and frontal ROIs, tracking language-related areas for reading and structuring. Greater exponents in temporo-parietal vs. frontal ROIs in L1, echoed in L2, suggest increased inhibitory regulation in temporo-parietal ROIs. We address the theoretical debate on the implementation of syntactic computations within the brain, considering asymmetries across L1 and L2. Stabilization is required in neural computations. Hence, increased exponents reflecting greater inhibition in temporo-parietal relative to frontal ROIs seem to align with a syntactic engagement. Offsets and exponents, as indices of neuronal spiking and excitatory–inhibitory balance, crucially point to differences in neural dynamics between cell assemblies in the processing of L1 and L2 French in reading, addressing less well-tuned cell assemblies for L2. Addressing L2 acquisition as a characteristic of a fully linguistic brain, we consider potential theoretical implications of these asymmetries based on current proposals on the nature of language computations and on the language-ready brain, suggesting hypotheses to guide future research.

## 2. Aims, Hypotheses, and Research Questions

### 2.1. Aims

Our overarching goal is to characterize how the language network adapts to the processing of L2 input [6,7,8,9,54] by building a novel neurofunctional subsystem for the L2 with its own activation thresholds [10]. In the current study, our first empirical research goal is to document differences in aperiodic activity across L1 and L2 suggestive of the engagement of a subnetwork implementing syntactic computations in language generation [75,76]. This would be consistent with inherent parsing-based constraints as new cell assemblies are recruited for L2 processing in L2 acquisition. Our second empirical research goal is to document differences in aperiodic activity between L1 and L2, suggesting distinct neurofunctional cell assemblies within a specialized adaptive network. Under the Full Transfer/Full Access [3] model, with L2 acquisition starting under an L1 initial state and guided by an FLN computational network accounting for UG, distinct aperiodic activity patterns for more English-like vs. less English-like French traits should arise in L2 French processing, but not in L1 French processing. Diminished offsets and exponents for more English-like vs. less English-like French traits would point to better-tuned pre-recruited vs. less well-tuned novel circuit modules in the cell assembly for L2.

To advance these goals, we examined L1 vs. L2 aperiodic activity during reading as *wh*-fillers in biclausal filler-gap dependencies are recursively processed. In the processing of (1a–d), the *wh*-filler *quelle décision* ‘which decision’ must be maintained in item memory and superposed on multiple re-representations through multiple phases of syntactic computations for the matrix and embedded clauses, before its eventual association with, and thematic integration within, the embedded clause. Since the *wh*-filler needs only to be pronounced once, recursive computations across clauses (CPs) typically implicate multiple silent copies or traces (marked as *t* in [1a–d]) of the *wh*-filler at intermediate (clause-edge) and thematic gap sites [_CP_ [_wh-DP_ *quelle nP*] … [_CP_ <*quelle nP*> … <*quelle nP*> …]]. The presence of such intermediate clause-edge copies is supported by the fact that in some language use, these copies can be pronounced—as documented in Afrikaans [77], child English [78], and German [79]—in presumed reactivation through externalization at the edge of a computational cycle. In typical silent cases, *wh*-filler re-representation at the clause-edge has been tied to increased processing load followed by less effortful filler integration in thematic position across L1 and L2 populations [75,80,81,82,83,84].

(1a) *Quelle décision le concernant est-ce que Paul a dit t que Lydie avait rejetée t sans hésitation?*
  Which decision him regarding is-it that Paul has said that Lydie had rejected without hesitation
  ‘Which decision regarding him did Paul say that Lydie had rejected without hesitation?’
(1b) *Quelle décision à propos de lui est-ce que Paul a dit t que Lydie avait rejetée t sans hésitation?*
  Which decision about him is-it that Paul has said that Lydie had rejected without hesitation
  ‘Which decision about him did Paul say that Lydie had rejected without hesitation?’
(1c) *Quelle décision à son sujet est-ce que Paul a dit t que Lydie avait rejetée t sans hésitation?*
  Which decision about him/her is-it that Paul has said that Lydie had rejected without hesitation
  ‘Which decision about him/her did Paul say that Lydie had rejected without hesitation?’
(1d) *Quelle décision à son sujet est-ce que Lydie a dit t que Paul avait rejetée t sans hésitation?*
  Which decision about him/her is-it that Lydie has said that Paul had rejected without hesitation
  ‘Which decision about him/her did Lydie say that Paul had rejected without hesitation?’

We address the nature of L1 vs. L2 neurofunctional subsystems by considering the aperiodic activity tied to differences between English and French grammar. The processing of (1a–d) requires associating gender features to nominal representations. In preverbal clitic pronouns (as in *le concernant* ‘him + regarding’; 1a) and in strong pronouns (as in *à propos de lui* ‘about him’; 1b), the gender of the pronoun is tied to the gender of the antecedent (i.e., *Paul* in [1a, b]). English exhibits similar agreement patterns. In contrast with English, however, gender features for pronouns and antecedents apply across animacy, so that *le* ‘it-masc’ is associated with *vélo* ‘bike-masc’ vs. *la* ‘it-fem,’ associated with *bicyclette* ‘bike-fem’ in (2a, b).

(2a) *Ce vélo, Marc le veut.*
  This bike-masc, Marc it-masc wants
(2b) *Cette bicyclette, Marc la veut.*
  This bike-fem, Marc it-fem wants
  ‘This bike, Marc wants it.’

Crucially, genitive clitics (e.g., *son* ‘his-her-masc’, *sa* ‘his-her-fem’, *ses* ‘his-her-plural’) as in *son vélo* ‘his-her-masc bike-masc,’ or *ses vélos* ‘his-her-plural bike-plural’—like determiners as in *ce vélo* ‘this-masc bike-masc’ or *ces vélos* ‘these bike-plural’—agree with the head noun across gender and number. Thus, both *son vélo* and *sa bicyclette* could be used to refer to a bike that belongs to either Marc or Marie.

In L1-English L2-French speakers, the acquisition of French genitive pronouns, in which the gender of the pronoun is associated with the head noun of the genitive structure rather than the gender of the antecedent, therefore involves additional complexity in the reassembly of lexico-grammatical features. Indeed, low-to-intermediate-level L1-English L2-French learners often provide antecedent-gender-specified substitutes such as *elle vélo or *sa vélo to signal *her bike*, or *il bicyclette, *lui bicyclette, *son bicyclette, or *la bicyclette de lui to signal *his bike*. In (1a–d), therefore, interpretable gender-antecedent features for *le*/*la* ‘him/her’ (1a), and for *lui*/*elle* ‘him/her’ (1b) contrast with (1c, d) where the uninterpretable gender feature of *son* ‘his-her’ agrees with the head noun *sujet* ‘subject.’ Beyond these input-specific gender-based and morphological differences, strong computational similarities involve lexically unselected verbal modifier structures (1a) as in *decision regarding*, and lexically selected prepositional noun complement structures (1b–d) as in *decision about*. The latter involves distinct syntactic structures [85,86,87,88,89,90] across languages.

### 2.2. Hypotheses

On the adaptive language network hypothesis, language is implemented by a specialized neurofunctional network that must provide a basic computational mechanism for merging basic elements into new syntactic objects across languages, together with lexico-grammatical specifications for each language. The activity in occipital and bilateral temporo-parietal and frontal ROIs, tracking language-related areas for reading and structuring, should echo aspects of the language network: FLN subnetworks for hierarchy/Merge within the larger FLB network for linearization/sequencing and contextual use in discourse/pragmatics. On the declarative-procedural memory hypothesis [91], in which L2 acquisition starts very differently based on purely declarative memory, better-tuned activity is expected in the temporo-parietal ROIs tied to declarative memory than in frontal ROIs tied to procedural memory. In contrast, on the adaptive network hypothesis, aperiodic components as measures of basic spiking and excitatory–inhibitory balance in L1 and L2 processing should echo the FLN/FLB structure of the greater language network. Hence, across L2 vs. L1 neurofunctional subsystems recruited by French input, differences between aperiodic components should therefore be modulated by ROIs echoing the FLN/FLB across L1 and L2 processing or a specific declarative-procedural structure in L2 processing.

Additionally, aperiodic components across ROIs reflecting FLB could reflect less well-tuned later-acquired circuit modules for L2 specifications—with possible modulation by L1-recruited circuit modules for grammatical features and lexico-grammatical elements for L2 French in L1 transfer. However, such differences may be small in a consolidated system, since advanced L1 English-L2 French learners come to master the use of head-noun-agreeing pronouns in the genitive structure.

Within the modern minimalist framework, language involves a non-associative commutative workspace temporarily holding manipulable basic elements and rooted trees, with a Hopf algebraic structure [25]. Trees for hierarchical structures are implemented through the recursive grafting of complex and elementary rooted units, implicating simultaneous complementary product (composition) and coproduct (decomposition) operations in the building of a complex object and the extraction of its elements, enabling extracted elements to be remerged [25]. The mathematical structure of Merge is presumed to reflect the biological nature of computational circuitry for Merge, possibly implicated by bilateral parietal neuron-to-neuron FLN computational complementary connections for product and coproduct, with a workspace enabled by cortical-subcortical connections [41,42]. As temporal, parietal, and pre-frontal cortices are implicated in language processing, a range of alternative hypotheses about the implementation of Merge/hierarchical syntax have been proposed [34,35,37,46,48].

Bilateral activity across the frontal gyrus, together with low parietal lobules in learning [44] echoed by increased parietal bilateral activity reflecting processing load increases in relative clauses in L1 processing [45], might best align with the current understanding of the nature of Merge within a Hopf algebra architecture [25], given complementary roles in hemispheres [92]. Under the temporo-parietal view [37,41,42,48], a bihemispheric lateralized temporo-parietal subnetwork for FLN would predict greater dynamics captured in temporo-parietal ROIs than in frontal ROIs in the recursive computation of *wh*-dependencies. Such a computational mechanism for the FLN [26] allows for the parsing of input in implicit grammatical learning in L1 [29] and L2 [28,75]: The extraction of basic elements in the parsing of new input allows for the long-term storage of lexico-grammatical specifications for basic elements in implicit grammatical learning. Such a basic recursive computational mechanism across languages empowers the infinity of language as a symbolic system and enables the recruitment of microlevel neuronal circuit modules for the long-term encoding of discrete basic lexico-grammatical specifications.

Thus, as neuronal microcircuit modules for basic lexico-grammatical elements are recruited in the parsing of L2-French input in L1-English L2-French acquisition, neuronal assemblies that were first recruited for L1 English must also respond to L1-shared traits of the L2 input (such as categories for the nominal and verbal systems). Circuit modules that are pre-recruited in L1 acquisition must then also be integrated with microcircuit modules for the gender system newly recruited in endogenous activation. L2 neurocognitive representations within an adaptive network that involves an already consolidated neurofunctional subsystem for L1 representations should result in spike patterns for new microcircuit modules dependent on synaptic plasticity relative to pre-existing circuits. Increased excitatory spiking activity in L2 processing would be consistent with increased BOLD signals modulated by L2 proficiency [7,8,9]. Inhibitory activity regulates the synaptic-plasticity-based building and consolidation of neuronal circuit modules for new features and basic lexico-grammatical specifications in the representation of language. Greater regulation for novel circuit modules predicts distinct inhibitory and excitatory balances in L1 vs. L2 processing, with increasing inhibitory signal ratio steepening the 1/f^x^ slope in L2. Aperiodic activity could still reflect somewhat greater regulation in advanced proficiency L1-English L2-French speakers than in L1-French speakers. These differences might also be modulated by the degree of similarity between English and French lexico-grammatical traits reflecting microcircuit overlap across subnetworks.

L1 vs. L2 excitatory–inhibitory balance differences between antecedent-gender-specified and antecedent-gender-unspecified pronouns in *wh*-fillers could therefore address aspects of the L2 subsystems as *wh*-DPs are recursively processed in Merge in re-representation. In addition to the selection of the noun *sujet* by the preposition *à* to express *about*, the greater complexity of the structure *à son sujet* ‘about him/her’ (1c, d) arises as a novel L2 micro-level neuro-representation for *son* ‘his-her-masc’ recursively interacts with computational circuitry in multiple cycles of *wh*-filler computations. Aperiodic activity differences for L2 vs. L1 French neurofunctional subsystems should be mitigated by more English-like structures (1a + b) vs. less English-like French-specific structures (1c + d).

### 2.3. Research Questions

Our study was guided by the following research questions, considering differences in aperiodic activity across L1 and L2, suggestive of the engagement of a subnetwork implementing syntactic computations in language generation as well as differences in aperiodic activity between L1 and L2, suggesting distinct neurofunctional cell assemblies within a specialized adaptive network:

RQ1: Will aperiodic offsets and exponents in biclausal *wh*-dependency processing show similar ROI asymmetries for L1 and L2 neural activity, consistent with temporo-parietal sites for FLN vs. larger FLB?

RQ2: Will aperiodic offsets and exponents across ROIs reveal L1–L2 differences, with potential modulation by degree of L1–L2 similarity, reflecting less well-tuned circuit modules in L2 and L1–L2 circuit overlaps?

Aperiodic activity shifts across ROIs and across L1 and L2 can address the cognitive status of the adaptive language network in terms of classical computational vs. connectionist approaches [33]. The creation of new concepts places language within a population-based model for neural circuitry in high-level cognition, with neuron-based systems relevant to sensorimotor phenomena, with classical symbolic computations implicating neuron-based dedicated circuits for low-level cognition in specific evolution [75]. Therefore, increased spiking and regulation in temporo-parietal ROIs (RQ1), suggesting the engagement of a neuron-based dedicated temporo-parietal computational subnetwork for syntax, would support a classical symbolic approach to higher-level cognition in presumed interaction with population-based representations.

Furthermore, L1 vs. L2 aperiodic asymmetries across ROIs (RQ2) implicating distributed cell assemblies recruited for L1 and L2 across domains (i.e., meaning, morphology, segmental phonology, prosody, spelling) would support a population-based connectionist approach. Asymmetries in levels of spiking and regulation reflecting both distributed cell assemblies recruited in L1 and L2 for basic representations and lexico-grammatical elements, together with presumed neuron-based dedicated circuits across L1 and L2 processing, would show both classical and connectionist theories as likely crucial to language cognition. This mechanism would capture parser-based language learning, with circuits dedicated to computations enabling the recruitment of distributed cell assemblies in target input.

## 3. Materials and Methods

This research was approved by the Indiana University Review Board. At the start of the experimental session, participants read the study’s Statement of Informed Consent. They were asked whether they had any questions and whether they consented to participate in the study. Written consent was waived due to the non-invasive nature of the study and to protect participant anonymity. Participants provided verbal consent to the researcher, in line with the approved IRB Protocol, and were reminded that they could withdraw at any point. After providing biographical information, participants completed a C-test to gauge their overall proficiency in French. A C-test involves paragraph-length texts in which the second half of every other word is removed after an initial sentence providing context. The C-test [93] consisted of two unrelated texts with 50 partially missing words (25 content words and 25 function words) across the two paragraphs. Respondents were given 10 min to fill in the missing words. C-tests were scored for accuracy out of 50 points. Finally, participants completed the EEG task, as described below; including breaks, the total task time was around 1 h. These procedures ensured that the subjects would not be fatigued and could be expected to stay engaged.

The stimuli consisted of 200 items, among which 100 critical items included 25 quadruples as in (1a–d), with 50 more English-like items in which the gender of the clitic or strong pronoun reflected the gender of the antecedent (1a, b), and 50 less English-like items in which the genitive pronoun agreed in gender with the head noun of the genitive structure rather than with the antecedent (1c, d). Of these second 50 items, 25 had a female antecedent in the matrix clause as in (1c), and 25 had a male antecedent as in (1d). Similarly, half of the more English-like items (1a, b) involved masculine pronouns *le* and *lui* ‘3p.sing.masc’ and half involved feminine pronouns *la* and *elle* ‘3p.sing.fem’. Crucially, antecedent-gender-specified pronouns *la* and *elle* and antecedent-gender-unspecified pronoun *son* all allow the retrieval of the matrix subject as the antecedent. The 100 distractor items involved complex interrogative structures and permutations like target items, counterbalanced so that no grouping stood out.

E-Prime [94] delivered the stimuli in a rapid serial visual presentation reading task. The stimuli appeared word by word at the center of the screen in a 36-point Consolas font, using normal orthographic conventions. They appeared in four blocks presented in random order. Within each block, stimuli were also presented in random order. Participants sat in a chair facing a computer monitor at a distance of approximately four feet. A fixation cross at the center of the screen preceded each item, lasting 700 ms. The task was found to be hard but manageable for advanced L2 speakers in stimulus preparation. Due to the time required for E-prime to load each word and for the monitor’s refresh rate, the total presentation time per word was 566 ms (300 ms presentation, 250 ms interstimulus interval, and a 16 ms refresh rate between words), accommodating L2 speakers without being unnaturally slow for L1 speakers.

Respondents were trained to read questions like the stimuli and then accept or reject follow-up comprehension statements, which were presented in their entirety for a maximum of 3500 ms. These comprehension checks were of several types: some examined the propensity for an anaphoric interpretation, while others queried other aspects of the sentences. Participants quickly responded to the statements by pressing the left arrow key for ‘Yes/True’ and the right arrow key for ‘No/False.’ There was a training session of six items, which could be repeated before moving on to the experiment. In the training, all items were followed by a comprehension statement; in the task, only two-thirds were. However, L1 and L2 speakers alike interpreted the pronoun as referring to the gender-matched noun phrase 70% of the time in critical stimuli. Naturally, a set of questions like our stimuli seems plausible in only a limited set of situations. Thus, respondents were introduced to a context involving two characters who were devoted followers of a television series. One of the characters, however, had missed some episodes and asked the other character questions to catch up.

Both L1 and L2 participants found the task challenging because the interpretation combines both *wh*-movement and anaphora. Hence, we limited stimuli to 100 critical items and 100 distractors. The number of trials per condition in the literature ranges widely, from 20 to 140, depending on the complexity of the structures investigated. The inherent variability among L2 speakers needs to be mitigated in L2 research, and L2 speakers also typically have a more limited vocabulary size. Hence, presenting all items in a set like (1a–d) to participants enables a truer representation of L2 grammatical processing ability, which might otherwise be diluted. No two items from a set ever appeared in the same block. These measures maximize the quality of group-level data.

### 3.1. Participants and Testing Procedures

Following Lewis et al.’s [95] examination of oscillations in 20 L1 speakers and 20 advanced L2 speakers, we selected a population sample of 48, with two groups of 24 English and French bilinguals. The 24 L1 speakers of French (20 right-handed, 4 left-handed; average age = 26.6, *SD* = 4.32) were tested in the US, where most were graduate students, exchange program participants, or visitors to campus. They had, on average, lived abroad for 2.4 years (*SD* = 2.61) at the time of testing. The average C-test score was 48.7/50, with a range of 45–50. The 24 L2 speakers of French (23 right-handed, 1 left-handed; average age = 28.8, *SD* = 6.37) began acquiring French during secondary schooling or later. These participants were graduate students and advanced undergraduate students in the US after a stay abroad at the time of testing. They had spent an average total length of stay of 1.2 years (*SD* = 0.69) in a Francophone country. C-test scores (average 45.5/50; range 33–50) clearly indicate advanced proficiency.

Bilingual experience is constant across groups. Both L1 and L2 speakers share the use of two languages at the time of testing. Therefore, this experimental set-up ensures that distinctions between L1 vs. L2 groups address early acquired L1 vs. later-learned L2 grammatical knowledge, rather than bilingual vs. monolingual differences. All participants had completed or were currently enrolled in postsecondary education. No history of dyslexia was reported. There were no exclusion criteria beyond unreasonably noisy EEG recordings (see next section) or neurological disorders.

### 3.2. EEG Procedures

**Figure 1:**
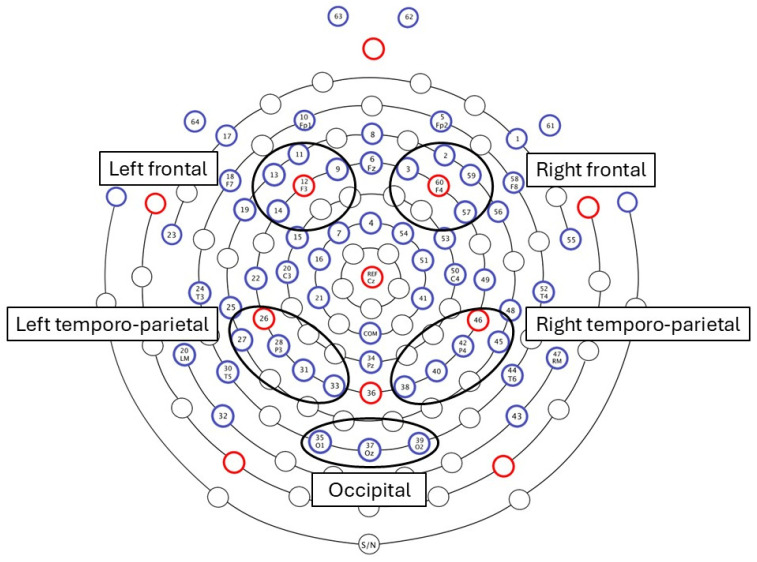
The EGI 64-electrode system with regions of interest highlighted.

EEG was recorded at a 1000 Hz sampling rate via a 64-electrode EGI system (Electrical Geodesics Inc., Eugene, OR, USA; as displayed in Figure 1, with ROIs indicated) referenced to Cz (vertex). The signal was collected using a Net Amps 300 amplifier with a gain of 5000 and acquisition software Netstation (version 4.5.4). Impedances were verified to be below 50 kΩ before each of the 4 blocks in the task. All preprocessing and data cleaning procedures were performed using the EEGLAB toolbox based on MATLAB version 9.5 [96]. An 8 ms latency shift due to the amplifier was corrected before preprocessing. Line noise was removed using the CleanLine plugin for EEGLAB [97]. The continuous data were then divided into 5.2 s epochs, starting with *est-ce que* (the question marker) and running to the end of the sentence. Following segmentation, we visually inspected each epoch for bad channels, and if a channel was bad in more than 10% of epochs, we removed the whole channel. It has been demonstrated that both cranial (i.e., face and neck) and ocular muscle activity can create noise in high-frequency EEG measures, which should thus be reported and interpreted with caution [98]. In the current study, aperiodic activity is reported up to 40 Hz. Hipp and Siegel [99] showed that removing such artifacts from the EEG recording by rejecting data sections affected by artifactual signals or Independent Component Analysis (ICA) can allow for more confident analysis of high-frequency EEG. Therefore, we visually inspected each epoch and systematically removed any epoch including any unexpected EMG activity (i.e., furrowing of the brow, face and neck movements, but not blinks). In line with the recommendations of [99], we used ICA to effectively remove the remaining ocular activity. Twelve subjects with greater than 10% bad channels or greater than 30% bad epochs were excluded from analysis, leaving the 24 L1 speakers and 24 L2 speakers described above. An average of 87% of trials was retained across subjects (*SD* = 2.26). The average number of trials retained was similar across groups (*SD* for L1 = 3.30, *SD* for L2 = 3.53, *p* = 0.648) and conditions: 21.4 (2a, *SD* = 2.32), 21.0 (2b, *SD* = 2.04), 21.3 (2c, *SD* = 2.22), and 21.4 (2d, *SD* = 2.46) (*p* = 0.786). For L1 speakers, 290 components were rejected, for an average of 12 per subject; for L2 speakers, 345 components were rejected, for an average of 14 per subject; and for the whole population, 635 components were rejected, for an average of 13 per subject. No significant difference arose between groups in the number of components removed (*p* = 0.35). All remaining trials were included in analyses. The data were average referenced, and missing channels were interpolated. Brain processing, as the participants computed a biclausal filler-gap dependency, is independent of their behavior on comprehension checks following the sentence.

### 3.3. Analytical Procedures

We used the Brainstorm toolbox [100] to analyze the aperiodic activity of 24 L1 and 24 L2 speakers. Preprocessed EEG data for two conditions (more English-like an-tecedent-gender marked pronouns [1a, b] vs. less English-like antecedent-gender unmarked pronouns [1c, d]) for these 48 subjects were loaded into the Brainstorm toolbox. Offsets and exponents were each analyzed in SPSS (version 31) with a linear mixed model (LMM) with condition (more and less L1-like items) and 5 ROIs (occipital, temporo-parietal, and frontal areas) as within-subject factors and L1–L2 group as a between-subject factor, and random intercepts for participant and electrode. SPSS provides tests of fixed effects, parameter estimates for the fixed effects, and Bonferroni-adjusted pairwise comparisons based on estimated marginal means. The time window of theoretical interest is constituted by the entire string following the *wh*-filler (*est-ce que Paul a dit que Lydie avait rejetée sans hésitation* ‘did Paul say that Lydie rejected without hesitation’), so that the aperiodic activity reported is associated with the same word string across conditions. It is therefore expected that condition effects would reflect abstract language processing.

## 4. Results

Although the LMMs for offsets and exponents did not provide main effects of L1–L2 group, offset: *F*(1, 46.029) = 1.657, *p* = 0.204; exponent *F*(1, 46.029) = 2.975, *p* = 0.091—aligning with the advanced proficiency of the L2 group—they did reveal very robust ROI*L1–L2 group interactions as well as main effects of ROIs. Hence, the LMM for offsets, correlated with neuronal population spiking, revealed a significant fixed effect of ROI, *F*(4, 19.387) = 3.393, *p* = 0.029, with an interaction of ROIs with L1–L2, *F*(4, 2126.337) = 31.595, *p* < 0.001. Likewise, the LMM for exponents, correlated with excitatory–inhibitory balance, revealed a significant fixed effect of ROIs, *F*(4, 19.695) = 9.278, *p* < 0.001, with an interaction of ROIs with L1–L2 groups, *F*(4, 2126.610) = 25.078, *p* < 0.001. Estimates of fixed effects for each ROI on offsets and exponents across L1 and L2 are provided in Table 1. Effects for offsets overall suggest significantly less neuronal population spiking captured by the electrodes of the frontal left hemisphere (ROI 1) and right hemisphere (ROI 3) relative to the occipital electrodes (ROI 5). The level of spiking captured by the temporo-parietal electrodes of the left (ROI 2) and right (ROI 4) hemispheres aligns with the level of spiking captured by the occipital electrodes (ROI 5). Effects for exponents reflecting regulation revealed similar asymmetries, with the electrodes of the frontal left (ROI 1) and right (ROI 3) hemispheres providing smaller exponents than the occipital electrodes (ROI 5). Crucially, exponent values captured by the temporo-parietal left (ROI 2) and right (ROI 4) hemisphere electrodes aligned with those captured by the occipital electrodes (ROI 5). These estimates highlight less aperiodic activity in the frontal areas across L1 and L2 with similar differences across hemispheres.

**Figure 2:**
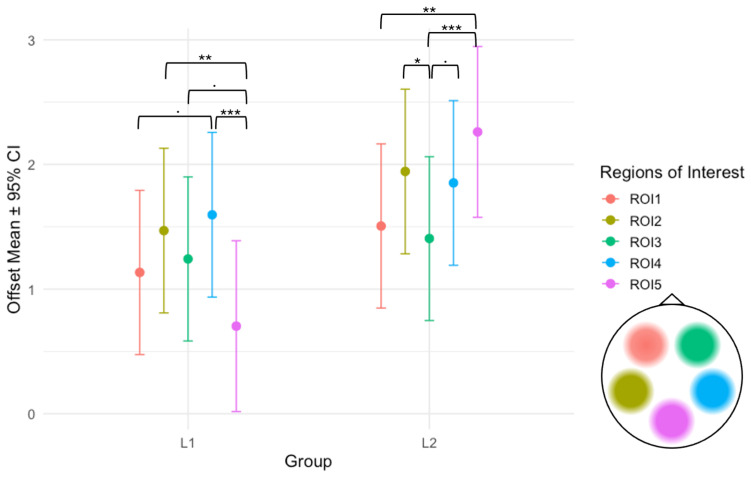
Mean offsets by ROI and L1–L2 group. Note. · *p* < 0.10. * *p* < 0.05. ** *p* < 0.01. *** *p* < 0.001.

**Figure 3:**
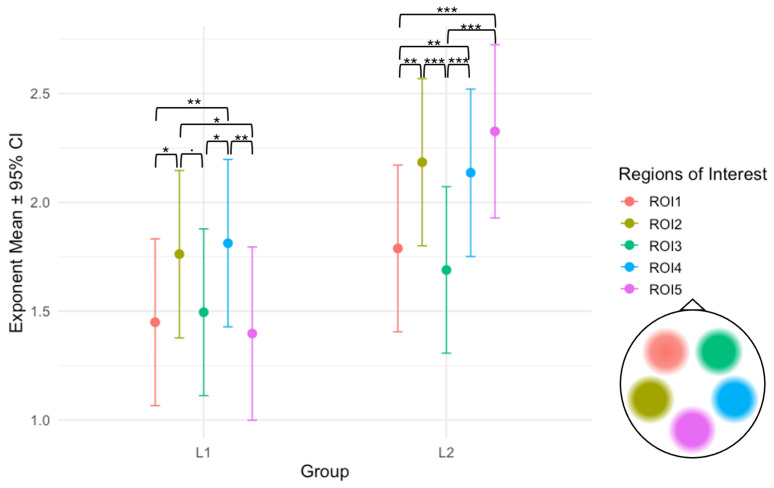
Mean exponents by ROI and L1–L2 group. Note. · *p* < 0.10. * *p* < 0.05. ** *p* < 0.01. *** *p* < 0.001.

Across offsets and exponents, univariate tests for the simple effects of ROI within L1 and L2 groups based on the linearly independent pairwise comparisons among the estimated marginal means revealed very similar strength of ROI effects, offset: L1: *F*(4, 25.860) = 6.829, *p* < 0.001; L2: *F*(4, 26.127) = 7.314, *p* < 0.001; exponent: L1: *F*(4, 26.190) = 7.574, *p* < 0.001; L2: *F*(4, 26.456) = 15.149, *p* < 0.001. Turning to the details of these ROI effects in L1 and L2 groups, Table 2 presents pairwise comparisons of ROIs based on offset values, while Table 3 presents pairwise comparisons of ROIs based on exponent values. Figure 2 and Figure 3 provide a visual depiction of the patterns for offsets and exponents, respectively, in L1 and L2 groups.

As shown in Figure 2 and Table 2, differences in offsets for L1 speakers revealed the ROIs over the temporo-parietal areas (ROI 2, ROI 4) to be significantly different from ROI 5 over the occipital region tied to visual input, indicating significantly greater spiking in the temporo-parietal areas. However, in the L2 group, offsets for the occipital ROI 5 were much more closely aligned with offsets in temporo-parietal ROIs (ROI 2, ROI 4) and distinct from frontal ROIs (ROI 1, ROI 3). This frontal vs. occipital ROI asymmetry in offsets in the L2 group, therefore, suggests greater neuronal spiking in the mapping of L2 spelling to (morpho)phonology, in contrast with the early established L1 signal.

As shown in Figure 3 and Table 3, exponent values in frontal ROIs (ROI 1, ROI 3) and in temporo-parietal ROIs (ROI 2, ROI 4) were statistically flat in L1-French speakers. Exponent values in temporo-parietal ROIs (ROI 2, ROI 4) were statistically greater than in the frontal ROIs and the occipital ROI, reflecting greater inhibitory activity detected by electrodes in temporo-parietal ROIs. These suggest increased circuit regulation across temporo-parietal ROIs in filler-gap processing.

In advanced L2 speakers, exponent values suggest greater regulation in temporo-parietal ROIs and in occipital ROIs than in frontal ROIs. Hence, across L1 and L2, statistically greater exponents were found in temporo-parietal ROIs (ROI 2, ROI 4) than in frontal ROIs (ROI 1, ROI 3). The frontal vs. temporo-parietal asymmetry across L1 and L2 aligns with a specific role for the activity of these cortical areas in language processing. The major difference between the patterns for L1 vs. L2 resides in the occipital ROI, with increased inhibitory activity in the EEG signal among the L2 group. This difference suggests greater regulation, presumably due to the management of the later developed written signal for L2 morphophonology.

The LMMs for offsets and exponents did not provide a main effect of Condition, offset: *F*(1, 2123.407) = 0.318, *p* = 0.573; exponent: *F*(1, 2123.407) = 0.411, *p* = 0.521, nor an interaction of Conditions with L1–L2 group, offset: *F*(1, 2123.407) = 0.194, *p* = 0.659; exponent: *F*(1, 2123.407) = 0.852, *p* = 0.356. Less L1-like traits, implicating a greater range of endogenously recruited circuit modules for L2 lexemes, were expected to yield somewhat greater offsets and exponents than more English-like traits in the L2 group, with no such differences in the L1 group. Given the potential theoretical relevance of L1–L2 overlap to assess L1 transfer as the initial state of L2 acquisition [3], we conducted follow-up *t*-test comparisons targeting more vs. less English-like traits within each group. These *t*-tests for offsets and exponents for less vs. more English-like traits across and within ROIs should be considered as purely descriptive and exploratory.

**Figure 4:**
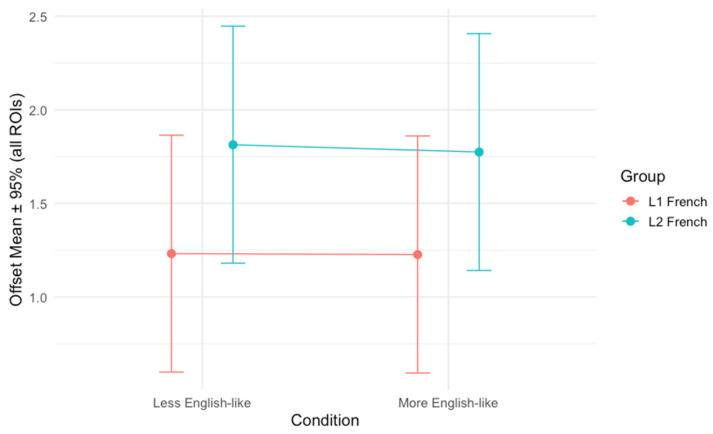
L1–L2 offset means for more and less English-like traits.

**Figure 5:**
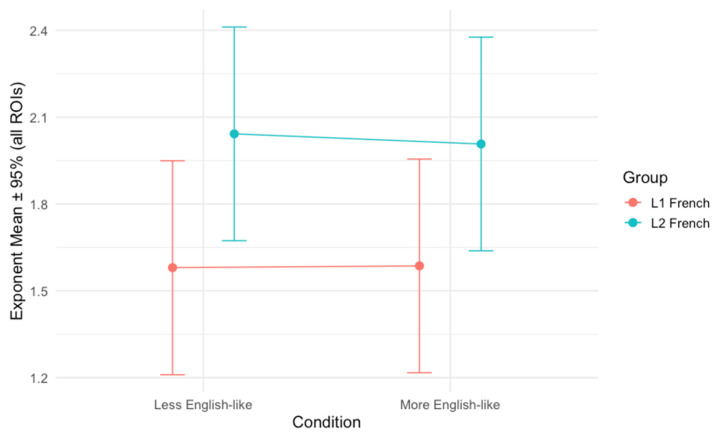
L1–L2 exponent means for more and less English-like traits.

As expected, the L1 group showed no differences between less vs. more English-like conditions across ROIs, offset: *t*(551) = 0.209, *p* = 0.417; exponent: *t*(551) = 0.873, *p* = 0.191. However, and also as theoretically expected from the point of view of L1 transfer, greater offset and exponent values for less English-like traits than more English-like traits were indeed found among the L2 group in this direct examination: offset: *t*(551) = 2.418, *p* = 0.016; exponent: *t*(551) = 3.393, *p* < 0.001. These patterns are visually represented in Figure 4 for offsets and in Figure 5 for exponents. As shown in Table 4, the same differences arose across the five ROIs. These effects were more robust in right frontal ROI 3, consistent with the location of ERP effects tied to anaphoric processing [101]. These purely descriptive effects, which failed to reach significance within the larger LMMs, potentially align with shared L1–L2 circuits in an L2 neurofunctional subsystem, with somewhat greater spiking and regulation associated with less L1–L2 overlap. Given this possibility in advanced L2 speakers, more robust effects of L1–L2 overlap would be expected at lower levels of L2 proficiency.

## 5. Discussion

Reading involves highly coordinated, left-lateralized circuitry that implicates pre-established circuits for vision, audition, and the lexicon linking the occipital, temporal, and frontal lobes [102]. Asymmetries in aperiodic activity in reading captured across occipital, temporo-parietal, and frontal ROIs can provide evidence about language processing, crucially addressing aspects of L2 acquisition. Different patterns of asymmetries across aperiodic components between ROIs for L1 vs. L2 suggest differences in regional neural engagement for L1 and L2 in the processing of written language input. In L1 speakers, offsets and exponents in the occipital ROI were significantly smaller than in the temporo-parietal ROIs. In L2 speakers, offsets and exponents in the occipital ROI matched those detected in the temporo-parietal ROIs. Across L1 and L2, the exponent values detected by the electrodes of the temporo-parietal ROIs differed from those detected by frontal ROIs. Offset values in L1 processing suggest a greater level of neuronal spiking in left and right temporo-parietal ROIs than in the occipital ROI, with frontal ROIs also revealing lower neuronal spiking levels. ROI asymmetries for exponent values in L1 also echoed these patterns, with activity captured by electrodes in left and right temporal-parietal ROIs suggesting a greater level of regulation than in frontal ROIs. Therefore, the aperiodic activity of L1 French speakers seems to reflect the structure of the language capacity as the written signal is mapped to morpho-phonological representations/the lexicon, and as abstract lexico-grammatical hierarchical structures are computed, together with linear phonological and prosodic representations and contextual interpretation. Increased neuronal spiking and inhibitory regulation detected in temporo-parietal ROIs relative to other ROIs therefore support differences in regional engagement that may reflect differences in the organization of underlying distributed cell assemblies in L1 vs. L2 processing. We now consider possible interpretations of these effects.

Addressing RQ1, greater aperiodic exponents were found in temporo-parietal ROIs vs. frontal ROIs in L1, echoed in L2, consistent with the engagement of dedicated areas for language during the recursive merging of *wh*-fillers in the computations of *wh*-dependencies [43]. This asymmetry echoes FLN computations within larger, distinct FLB distributed cell assemblies for L1 and L2. A body of neurolinguistic research has suggested bare syntax to be implemented in the left hemisphere pIFG, with temporo-parietal areas recruited for complex structures [35,36]. Using pseudo-Chinese characters in an artificial grammar paradigm, Liu et al. [36] considered sentential strings such as *blue ship sinks* in English or *navire bleu coule* ‘ship blue sinks’ in French, contrasting with nominal word salad strings *blue ship apples or *navire bleu pomme ‘ship blue apple.’ Sentential strings like *blue ships sink* require clause structure domains in which the nominal structure of *blue ships* is linked to a sentential subject/topic position and to a thematic position implicating recursive Merge. The authors reported frontal effects, including pIFG for sentential vs. word salad strings with no temporal lobe effects, attributing them to basic Merge. However, linearization and grammatical assessment in externalization cannot be excluded from these pIFG effects.

Crucially, the repeated merging of *wh*-fillers across clauses highlights recursion—together with significant working memory—as a fundamental characteristic of language. Claims of different areas for processing simple vs. complex structures in recursion is viewed as highly problematic: “In all existing theories of grammar, syntactic mechanisms are identical between ‘simple’ and ‘complex’ sentences (i.e., complex sentences may involve an increased number of iterations of these operations, but not the application of qualitatively different principles). Thus, there is no theoretical linguistic basis for the dichotomy between simple and complex syntax” [48] (p. 69). This argument against the pIFG area as implementing Merge also suggests the implementation of hierarchical computations for Merge “elsewhere” [51] (p. 2). On an alternative view of cortical involvement in basic syntax, therefore, the IFG is associated with externalization, while temporo-parietal sites are associated with abstract computations [37,41,48,51]. The greater visibility of the temporo-parietal engagement captured in the current study is presumably enhanced by a role in the recursive merging of *wh*-fillers.

Addressing RQ2, we did not find statistically significant main effects between L1 and L2, given that the L2 speakers are of advanced proficiency. L1–L2 group*ROI interactions, however, still suggest somewhat distinct aperiodic activity for consolidated L1 vs. L2 cell assemblies in advanced proficiency L2 speakers. These patterns indicate significant increases in spiking and inhibitory activity detected by the electrodes in the occipital ROI in L2. The observed differences between the aperiodic activity for L1 and L2 French captured by occipital electrodes, suggesting greater neuronal spiking and regulation in L2 reading, support the endogenous recruitment of circuit modules to a new mapping of the written signal to sound structure for L2. The LMMs also did not capture any significant aperiodic activity differences between less vs. more English-like traits in L2 vs. L1, presumably reflecting the advanced proficiency of L2 speakers. Hence, similar levels of activity across L1 and L2 and across less vs. more English-like traits in L2 seem aligned with the ongoing consolidation of novel circuit modules for L2 representations.

Surprisingly, therefore, a targeted investigation of these data also showed descriptive asymmetries that aligned with theoretical expectations in L1 transfer as an initial state of learning. Thus, somewhat greater neuronal spiking and inhibitory regulation were indicated by differences found in combined ROIs for less English-like *à son sujet* ‘about him-her’ stimuli than for more-English-like antecedent-gendered pronouns in *à propos de lui* ‘about him’ and *le concernant* ‘regarding him’ in L2 speakers only. Crucially, the absence of the same effect among L1 speakers rules out an argument that the asymmetries found in the L2 group were due to crosslinguistic influence (CLI) in syntactic parsing [103]. CLI would affect both L1 English-L2 French and L1 French-L2 English bilinguals; thus, the asymmetries in the L2 group instead likely point to an L1 transfer effect somewhat maintained through consolidation of the L2 neurofunctional subsystem in advanced learners. Such potential asymmetries will warrant further examination in earlier learning stages. Asymmetries in the right frontal ROI would also align with the distribution of referential ERP effects [104]. Aperiodic activity across proficiency levels in L2 acquisition could, therefore, provide new insight into the learning mechanisms.

Greater spiking and regulation in the L2 group, reflected by aperiodic activity differences in electrodes in the occipital ROI, suggest greater activity in information extraction from the written input signal. This effect aligns with delayed ERPs in slower reading by L2 speakers with otherwise maintained processing [105,106]. The aperiodic activity in the occipital ROI is therefore consistent with the possibility of partially distinct distributed cell assemblies for L1 and L2. New cell assemblies require the endogenous recruitment of cells into new circuit modules for elemental representations, implicating greater synaptic consolidation in learning. On Ullman’s declarative-procedural model for L2 acquisition [107,108], L2 development initially involves the declarative memory system in explicit learning. Unconscious automatic processes in syntax encoded in procedural memory arise later, with the implicit procedural mechanism gradually expanding and taking over from declarative memory. Hence, lexical N400 ERP effects are found first in L2 acquisition, although delayed, while morphosyntactic P600 effects arise later in L2 learners and are often also delayed and/or weaker [109,110]. Under the declarative-procedural model, therefore, L2 activity should originate in the temporo-parietal areas tied to declarative memory, with procedural processing in frontal areas established later. Thus, if L2 grammatical development initially involved the declarative memory system, these earlier established circuits within the declarative memory system should be better tuned than the newer ones within the procedural memory system. A declarative memory system for lexical information that can be consciously retrieved is certainly involved in explicit vocabulary learning.

Nevertheless, addressing the relative role of explicit vs. implicit learning in the acquisition of grammar, VanPatten and Smith [111] argued that the role of explicit learning in L2 acquisition seems to be much smaller than often thought. Similarities in aperiodic activity patterns in frontal vs. temporo-parietal ROIs across L1 and L2 best align with an adaptive language network. Increased exponents in temporal-parietal ROIs consistent with greater regulation in *wh*-dependency processing across L1 and L2 suggest the engagement of temporo-parietal areas in syntax. An adaptive language network allowing the parsing of new input therefore seems to best account for L2 sentence processing and acquisition as implicit knowledge over the long term [6]. Increased exponents detected in the engagement of temporo-parietal regions across distinct cell assemblies for L1 and L2 could mean dedicated streams interacting with population-based circuit modules for lexico-grammatical elements and their externalization in L1 and L2 processing. L1 vs. L2 asymmetries across ROIs, together with less vs. more L1-like L2 traits (RQ2), fit a connectionist model of overlapping distributed cell assemblies recruited for L1 and L2. This interpretation of findings, therefore, brings together alternative views of cognition [33], with interactions between Sherringtonian (neuron-based) and Hopfieldian (population-based) cognitive models for neural circuitry.

Patterns of excitatory–inhibitory balance suggested by exponents measured in electrodes in the temporo-parietal ROIs relative to frontal ROIs in L2, echoing the L1 pattern, support the engagement of temporo-parietal areas in filler-gap dependencies. The asymmetrical patterns for L2 including the occipital ROI also suggest much greater cognitive activity required to map the written input signal to morphophonology in L2. Hence, differences between the L1 and L2 groups suggest partially distinct cell assemblies for L1 and L2. In Hebbian learning, population-based circuit modules for basic lexico-grammatical elements and their externalization are created by intrinsic excitability changes in cell recruitment by L2 input. Synaptic connections are then developed and consolidated in later stages [22]. The recruitment of cells into new circuit modules in intrinsic excitability changes initially predicts less synchronous activity. Synaptic plasticity in the building and consolidation of circuits for L2 processing involves increased neuronal spiking and regulation. Somewhat increased aperiodic components still echoed in L2 vs. L1 are compatible with the synaptic development and consolidation of new dendrite branches rewiring neural circuits for L2, placing advanced proficiency in the consolidation phase. Crucially, the differences between frontal and temporo-parietal aperiodic activity in L1 speakers, echoed in the L2 group, and the differences across the same linear strings for more vs. less English-like conditions in L2 processing are consistent with a basic mechanistic FLN subnetwork dedicated to recursive computations in *wh*-filler integration.

In sum, the asymmetric distribution of aperiodic activity across L1 and L2 provides support for the FLN/FLB model of the adaptive language network, with a temporo-parietal mechanistic FLN subnetwork for syntactic computations. In the FLN/FLB generative model, language acquisition—as the recruitment of cells into population-based circuit modules for abstract lexemes across syntax and semantics and for their externalization in language use within the larger FLB—is constrained by an FLN computational subnetwork guiding the processing of language. On this view, L2 acquisition in implicit learning involves the recruitment of population-based circuit modules within an L1-tuned FLB. This recruitment updates preexisting circuit modules for basic L2 neuro-representations within an L1-tuned additive language network. It is constrained by a computational subnetwork for FLN, consistent with a full parse approach to the language acquisition device [112]. Under the Full Transfer/Full Access L2 acquisition account [3], L1-transfer effects could be attributed to the Hebbian learning mechanism, with UG constraints reflecting a computational subnetwork for FLN. The engagement of temporo-parietal regions echoes bilateral parietal fMRI effects reported in the processing of filler-gap dependencies in Chinese [45]. Bilateral temporo-parietal activity is often described in terms of cognitive control in the management of complex computations, rather than basic language computations. Cognitive control crucially also implicates frontal areas. Frontal vs. temporo-parietal asymmetries across L1 and more cognitively taxing L2, therefore, suggest similar processes in L1 and L2. We note that bilateral aperiodic patterns echo bilateral fMRI effects in filler-gap dependency processing. Growing evidence tying the IFG to the various steps needed in externalization [51] indirectly supports potential roles of temporo-parietal cortices in hierarchical computations [48], since syntax must happen “elsewhere” [51] (p. 2).

### Brain Science Extended Discussion

We now consider possible theoretical interpretations of the empirical finding of the engagement of temporo-parietal areas in *wh*-dependency computations in L1 and L2 French to guide future research. Following [26], we highlighted exponent asymmetries across L1 and L2 (RQ1) with inhibition in temporo-parietal ROIs—consistent with stabilization in neural computations across temporo-parietal cortices—reflecting a computational FLN vs. FLB distinction in the capacity for language. Highly specific asymmetries across L1 and L2 neurofunctional subsystems for French, which were acquired in very different contexts (tied to early childhood for L1 and post-puberty for L2) could reflect innate circuits tied to complementary functions arising in brain evolution [113]. For Sherringtonian circuits dedicated to computations to be feasible, specific complementary functions in computations should align with language evolution.

We, therefore, first consider possible functions implemented in the left and right temporo-parietal lobes addressing the processing of questions. Reviewing neurolinguistics studies, Bookheimer [92] pointed out that both hemispheres have crucial complementary roles in language processing. Activity across hemispheres highlights distinct complementary functions in language processing in terms of both sound structure and meaning. In the frontal lobe, the left hemisphere is tied to segmental phonetics, with the right hemisphere tied to prosodic sound structure. Thus, “prosody in speech comprehension encompasses a range of features, including intonations relevant to emotion, importance (e.g., stress or accents), and linguistic forms (questions vs. imperatives) that are associated with right hemisphere function” [92] (p. 179). The stimuli used in the current study involve *wh*-questions; we can, therefore, expect left and right activity linked to internal segmental and prosodic sound structure for questions in silent reading [114]. Within the temporo-parietal lobes, the left hemisphere is tied to lexical and compositional semantics, whereas temporo-parietal sites in the right hemisphere are associated with contextual interpretation and pragmatics, including formulaic language beyond lexical composition. “The right hemisphere makes a substantial contribution to many aspects of language comprehension, though not at the single-word level … lack[ing] both the one-to-one mapping of information with words and the sequential [compositional] analysis” [92] (p. 182). Meanings beyond compositionality can involve a range of formulaic uses from idioms such as *kick the bucket* as an expression for *DIE* together with contextual and figurative meaning for an expression like *The world is a stage*. In this experiment, compositional interpretations for these questions fully align with their discourse integration. Given effects reflecting activity during recursive computations, bilateral engagement across distinct regions tied to complementary functions raises the theoretical question of whether distinct complementary functions might also be involved in syntax as the recursive creation of rooted trees.

Advances in syntactic theory suggest a theoretical explanation of potential bihemispheric engagement in morphosyntax. The mathematics of syntax as a Hopf algebra [25] crucially involves dual complementary inverse product (⊗) and co-product (∆) functions for tree-based representations. Hence, ⊗ “combines workspaces [as tree-based vector spaces (V)] by taking their union” [25] (p. 6), with Merge(a, b) implicating the grafting (∧) of subtrees held in this workspace [a, b] into a rooted tree T, with T = a ∧ b. In contrast, ∆ “provides all the possible extractions of admissible terms” [25] (p. 6) with ∆(T) extracting subunits, which enables the re-merging of accessible subtrees in recursion. Crucially, a Hopf algebraic architecture is consistent with Murphy’s neurofunctional ROSE model for syntax based on oscillatory dynamics as a neural code [41,42]. Hence, gamma/delta coupling links gamma-implemented basic operations to complex structures in structural memory. Gamma/theta coupling implements item memory for basic/accessible elements. Theta/delta coupling integrates item and structural memory. Thus, converse cortico-cortical streams between bilateral representationally entangled coactivations for combined (a ∧ b) and separately maintained (a ⊔ b) tree-based elements would support inverse elemental operations in syntax. Cortico-subcortical connections would support workspaces for basic/accessible elements and for structural memory for rooted trees as vertices nested into a root vertex through Merge. Therefore, the implementation of complementary functions by neuron-to-neuron streams across lateralized temporo-parietal cortices, reported in a range of studies, together with subcortical connectivity, seems highly consistent with the mathematics of syntax.

The nature of L1 and L2 neurofunctional subsystems could help understand aspects of language readiness in the brain, enabling L2 acquisition. The possible engagement of temporal and parietal regions in Sherringtonian circuitry for syntactic processing [48]—interacting with the thalamus [51] in the implementation of elemental functions and working memory—would suggest circuits as “computationally dedicated modules that reflect the outcome of selective evolution” [113] (p. 367). This aligns, therefore, with neuron-to-neuron circuits dedicated to syntax in parsing. On this view, temporo-parietal engagement in syntactic computations should be consistent with the source of human-specific biological readiness for language.

Boeckx and Benítez-Burraco [27] tied a language-ready brain within the fully linguistic brain to its globularity [115] due to a substantial development of subcortical areas with the enhancement of cortico-thalamic connectivity, boosting language learning and use in modern humans. In contrast, the tent-like neanderthal brain was “not language-ready, *at least not in the way or to the extent in which sapiens’ brains are*. [emphasis added] This, of course, does not mean that Neanderthals … had no syntactic abilities at all” [27] (p. 3). Anatomic comparisons also support the role of self-domestication, associated with greater docility, cooperation, and reduced reactive aggression in language learning [12]. Computational ability is therefore required for language, but language acquisition and use are boosted by strong social skills in a fully linguistic brain. Hence, strong reactivity in autism spectrum and reactive attachment disorders leads to delayed language acquisition or non-verbalism [40]. In L2 acquisition, social integration also plays a significant role in L2 performance. Returning to bilateral effects, according to the bihemispheric theory [116,117,118,119,120], language evolution also implicates the separation of hemispheric functions in asymmetric development. Crucially, language-related mental psychosis can be tied to delayed cerebral lateralization. The separation of hemispheric functions in evolution therefore aligns with a dedicated bihemispheric computational subnetwork for Merge, implicating complementary basic operations.

In sum, the inherent capacity for L2 acquisition, as the recruitment of circuit modules for new features and basic lexico-grammatical elements in parsing, could reside in interactions with a dedicated computational mechanism for syntax. Communicative ability driven by social interactions enhances language production for a new input within a fully linguistic brain. Given the mathematics of syntax and the complementarity of hemispheric roles, such an engagement might possibly implicate cortico-cortical connections in temporo-parietal areas for complementary product and coproduct functions together, allowing recursion, with cortico-thalamic connections for workspaces in item and structural memory. These elemental functions for syntactic operations would arise in evolution in the separation of hemispheric roles in lateralization. This pattern would account for similar systems acquired in early childhood L1 and in post-puberty L2. Hence, increased regulation in temporo-parietal areas across L1 and L2, consistent with computational regulation across areas reflecting dedicated streams for syntax, potentially aligns with current theoretical understanding of syntax, offering a vision of brain activity that favors language-ready views of the biology of language.

## 6. Conclusions

Aperiodic components have been tied to childhood development, aging, consciousness, and task processing, reflecting changes in connectivity. Aperiodic activity addressing connectivity in the interpretation of *wh*-dependencies in L1 and L2 French during reading reveals distributed cell assemblies in L1 and L2 processing, with potential overlap between L1 and L2 circuit modules for basic representations in L2 processing. Crucially, across L1 and L2, temporo-parietal ROIs in both hemispheres seem highly engaged in *wh*-dependency processing, with stronger regulation consistent with stabilization in neural computations. Greater aperiodic exponents across L1 and L2 in temporo-parietal ROIs in *wh*-filler-gap dependency processing suggest computations across temporo-parietal cortices [48]. These temporo-parietal vs. frontal ROI asymmetries in aperiodic activity align with a similar mechanism across L1 and L2 echoing the FLN/FLB distinction. These aperiodic activity patterns align with behavior-based research on L2 acquisition showing Full Transfer/Full Access [3] with L1 providing an initial state and with additional features involving the endogenous recruitment of cells into new circuit modules. On this view, therefore, the generative capacity for natural language involves neuron-based circuitry dedicated to computations, which occurred in evolution. Neuron-based computational circuitry interacting with population-based neural circuits for basic representations, therefore, blends Sherringtonian and Hopfieldian views of cognition [33].

The activation of the temporo-parietal bilateral regions in syntax is still highly debated and constitutes a major challenge to fully resolve. Similar bilateral levels of aperiodic activity echo fMRI effects across hemispheres in syntactic processing [44,45]. Recursive merging as a fundamental property of language provides no reason for a different mechanism for complex structures [48,51]. Bilateral activity in syntactic computations still raises the question of the source of these effects. We therefore considered a range of complementary roles in sentence processing, including syntactic computations. Current theoretical understanding of syntax [25] provides “formal operations that are, ideally, elemental and generic” [121] (p. 285) and could therefore provide future greater insights into the neuroscience of language. Complementary operations in syntax highlight the possibility of bilateral connections across lateralized temporo-parietal cortices dedicated to syntax, given complementary roles for hemispheres. This complementarity aligns with bihemispheric views of language evolution, offering potential insights for further research.

## Figures and Tables

**Table 1:** Estimates of fixed effects for offsets and exponents.

Parameter	Estimate	Std. Error	df	*t*	*p*	95% CI
Offsets						
ROI 1	−0.755	0.208	40.065	−3.624	<0.001 *	[−1.176, −0.334]
ROI 2	−0.302	0.210	37.204	−1.439	0.159	[−0.728, 0.123]
ROI 3	−0.828	0.207	40.678	−4.003	<0.001 *	[−1.246, −0.410]
ROI 4	−0.421	0.210	37.204	−2.003	0.053	[−0.847, 0.005]
ROI 5						
Exponents						
ROI 1	−0.558	0.119	40.377	−4.698	<0.001 *	[−0.798, −0.318]
ROI 2	−0.152	0.120	37.470	−1.268	0.213	[−0.395, 0.091]
ROI 3	−0.643	0.118	40.983	−5.458	<0.001 *	[−0.882, −0.405]
ROI 4	−0.209	0.120	37.470	−1.744	0.089	[−0.452, 0.034]
ROI 5						

Note. ROI 1 = left frontal; ROI 2 = left temporo-parietal; ROI 3 = right frontal; ROI 4 = right temporo-parietal; ROI 5 = occipital. * *p* < 0.05 (with Bonferroni correction).

**Table 2:** Offsets pairwise ROI comparisons.

Comparison	Mean Difference	Std. Error	df	*p*	95% CI
L1 group					
ROI 1–ROI 2	−0.336	0.160	25.705	0.457	[−0.827, 0.155]
ROI 1–ROI 3	−0.108	0.153	35.817	1.000	[−0.566, 0.349]
ROI 1–ROI 4	−0.463	0.160	25.705	0.076	[−0.954, −0.028]
ROI 1–ROI 5	0.431	0.185	25.121	0.284	[−0.139, 1.001]
ROI 2–ROI 3	0.227	0.160	25.705	1.000	[−0.264, 0.718]
ROI 2–ROI 4	−0.127	0.162	23.527	1.000	[−0.630, 0.375]
ROI 2–ROI 5	0.767 *	0.187	23.527	0.004 *	[0.187, 1.347]
ROI 3–ROI 4	−0.354	0.160	25.705	0.358	[−0.846, 0.137]
ROI 3–ROI 5	0.539	0.185	25.121	0.075	[−0.031, 1.110]
ROI 4–ROI 5	0.894 *	0.187	23.527	<0.001 *	[0.314, 1.474]
L2 group					
ROI 1–ROI 2	−0.437	0.160	25.705	0.112	[−0.928, 0.054]
ROI 1–ROI 3	0.101	0.148	37.686	1.000	[−0.341, 0.543]
ROI 1–ROI 4	−0.345	0.160	25.705	0.405	[−0.836, 0.146]
ROI 1–ROI 5	−0.755 *	0.185	25.121	0.004 *	[−1.325, −0.184]
ROI 2–ROI 3	0.538 *	0.158	25.972	0.021 *	[0.054, 1.022]
ROI 2–ROI 4	0.092	0.162	23.527	1.000	[−0.411, 0.594]
ROI 2–ROI 5	−0.318	0.187	23.527	1.000	[−0.898, 0.263]
ROI 3–ROI 4	−0.446	0.158	25.972	0.090	[−0.930, 0.038]
ROI 3–ROI 5	−0.855 *	0.184	25.300	<0.001 *	[−1.420, −0.291]
ROI 4–ROI 5	−0.409	0.187	23.527	0.391	[−0.990, 0.171]

Note. ROI 1 = left frontal; ROI 2 = left temporo-parietal; ROI 3 = right frontal; ROI 4 = right temporo-parietal; ROI 5 = occipital. * *p* < 0.05 (with Bonferroni correction).

**Table 3:** Exponents pairwise ROI comparisons.

Comparison	Mean Difference	Std. Error	df	*p*	95% CI
L1 group					
ROI 1–ROI 2	−0.313 *	0.091	26.027	0.020 *	[−0.593, −0.033]
ROI 1–ROI 3	−0.046	0.087	36.411	1.000	[−0.307, 0.214]
ROI 1–ROI 4	−0.363 *	0.091	26.027	0.005 *	[−0.643, −0.084]
ROI 1–ROI 5	0.052	0.106	25.430	1.000	[−0.273, 0.377]
ROI 2–ROI 3	0.267	0.091	26.027	0.071	[−0.013, 0.547]
ROI 2–ROI 4	−0.050	0.093	23.804	1.000	[−0.337, 0.236]
ROI 2–ROI 5	0.365 *	0.107	23.804	0.023 *	[0.034, 0.695]
ROI 3–ROI 4	−0.317 *	0.091	26.027	0.018 *	[−0.597, −0.037]
ROI 3–ROI 5	0.098	0.106	25.430	1.000	[−0.227, 0.423]
ROI 4–ROI 5	0.415 *	0.107	23.804	0.007 *	[0.084, 0.746]
L2 group					
ROI 1–ROI 2	−0.396 *	0.091	26.027	0.002 *	[−0.676, −0.116]
ROI 1–ROI 3	0.099	0.085	38.292	1.000	[−0.153, 0.350]
ROI 1–ROI 4	−0.348 *	0.091	26.027	0.008 *	[−0.628, −0.068]
ROI 1–ROI 5	−0.538 *	0.106	25.430	<0.001 *	[−0.863, −0.213]
ROI 2–ROI 3	0.495 *	0.090	26.289	<0.001 *	[0.219, 0.771]
ROI 2–ROI 4	0.048	0.093	23.804	1.000	[−0.238, 0.335]
ROI 2–ROI 5	−0.142	0.107	23.804	1.000	[−0.473, 0.189]
ROI 3–ROI 4	−0.446 *	0.090	26.289	<0.001 *	[−0.722, −0.170]
ROI 3–ROI 5	−0.636 *	0.105	25.605	<0.001 *	[−0.958, −0.315]
ROI 4–ROI 5	−0.190	0.107	23.804	0.882	[−0.521, 0.141]

Note. ROI 1 = left frontal; ROI 2 = left temporo-parietal; ROI 3 = right frontal; ROI 4 = right temporo-parietal; ROI 5 = occipital. * *p* < 0.05 (with Bonferroni correction).

**Table 4:** L2 participants’ offsets and exponents for more- and less-English-like traits across ROIs.

ROI	More English-like		Less English-like		Difference		*t*	*df*	*p*
	Mean	*SD*	Mean	*SD*	Mean	*SD*			
Offsets									
1	1.45873788	1.306456920	1.48464124	1.306456920	−0.025903357	0.270090660	−1.051	119	0.296
2	1.91477843	1.976150674	1.97234826	2.002439597	−0.057569833	0.481269175	−1.310	119	0.193
3	1.39630357	1.812863708	1.47871953	1.800642620	−0.082415962 *	0.308919041	−2.923	119	0.004 *
4	1.84956234	2.012883877	1.85373072	2.093353688	−0.004168375	0.343870907	−0.133	119	0.895
5	2.24751056	1.702569280	2.27470958	1.985075241	−0.027199028	0.572233185	−0.403	71	0.688
Exponents									
1	1.76671673	0.873251509	1.78360163	0.911108043	−0.016884908	0.181520799	−1.019	119	0.310
2	2.16554206	1.203833569	2.20261918	1.250754086	−0.037077119	0.270087339	−1.504	119	0.135
3	1.67948282	1.044983745	1.72282774	1.073521045	−0.043344921 *	0.175588924	−2.704	119	0.008 *
4	2.12590997	1.235431103	2.14551373	1.256256837	−0.019603765	0.214398043	−1.002	119	0.319
5	2.29711028	1.121552789	2.35454147	1.238056006	−0.057431197	0.308308678	−1.581	71	0.118

Note. ROI 1 = left frontal; ROI 2 = left temporo-parietal; ROI 3 = right frontal; ROI 4 = right temporo-parietal; ROI 5 = occipital. * *p* < 0.05 (with Bonferroni correction).

## Data Availability

The original data presented in the study are openly available in IU DataCore at https://datacore.iu.edu/concern/data_sets/n870zs23t?locale=en (accessed on 4 January 2026).

## Abbreviations

The following abbreviations are used in this manuscript:

L1, L2	first language, second language
ROI	region of interest
FLN	language faculty in the narrow sense
FLB	language faculty in the broad sense
ERP	event-related potential
EEG	electroencephalography
IFG	inferior frontal gyrus
ICA	independent component analysis
LMM	linear mixed model
CLI	crosslinguistic influence
